# EPIGENETIC AGING AND XENOBIOTIC METABOLISM IN THE MOUSE LIVER

**DOI:** 10.1093/geroni/igae098.3469

**Published:** 2024-12-31

**Authors:** Sara Abudahab, Dalia Al Saeedy, Ola AlAzzeh, Joseph McClay

**Affiliations:** UST Global, Richmond, Virginia, United States; Virginia Commonwealth University, Richmond, Virginia, United States; Virginia Commonwealth University, Richmond, Virginia, United States; Virginia Commonwealth University, Richmond, Virginia, United States

## Abstract

Hepatic xenobiotic metabolism declines with age, and while intact metabolism is associated with longevity, few studies have explored the genome-wide impact of epigenetic aging on these processes. In this study, we used liver tissues from male C57BL/6 mice (4, 24, and 32 months), obtained from the National Institute on Aging. We mapped DNA methylation changes using Reduced Representation Bisulfite Sequencing (RRBS) to identify age-associated differentially methylated sites (a-DMS). Our findings demonstrate significant age-associated methylation changes, with younger mice (4 months) displaying different patterns compared to older counterparts. First, we found that genes encoding Phase I and II drug-metabolizing enzymes, in addition to ABC and SLC transporters, showed differential methylation with age. Targeted analysis of important pharmacogenes (Cyp1a2, Cyp2d9, and Abcc2) showed that aging led to Cyp2d9 hypermethylation and reduced expression, while Abcc2 expression remained unchanged. Cyp1a2 was hypomethylated with age, yet its expression decreased. Bioinformatic analysis revealed that a-DMS hypomethylated with age were enriched for HNF4α binding sites. HNF4α is downregulated with age, potentially explaining the reduction in Cyp1a2 expression despite hypomethylation. Pathway analysis of a-DMS revealed disruptions in xenobiotic metabolism pathways, including small molecule catabolism, are evident in both normally aged (24 months) and super-aged (32 months) mice when compared to the younger group (4 months). This study underscores the impact of epigenetic modifications in aging on liver function and xenobiotic metabolism.

